# Depressive Symptoms and Quality of Life in Patients With Heart Failure and an Implantable Cardioverter-Defibrillator

**DOI:** 10.3389/fpsyt.2022.827967

**Published:** 2022-06-17

**Authors:** Christos Zormpas, Kai G. Kahl, Stephan Hohmann, Hanno Oswald, Christopher Stiel, Christian Veltmann, Johann Bauersachs, David Duncker

**Affiliations:** ^1^Hannover Heart Rhythm Center, Department of Cardiology and Angiology, Hannover Medical School, Hannover, Germany; ^2^Department of Psychiatry, Social Psychiatry and Psychotherapy, Hannover Medical School, Hannover, Germany; ^3^Department of Cardiology, Pneumology, Angiology and Intensive Care Medicine, Klinikum Peine, Peine, Germany; ^4^Department of Neurology, University Hospital Schleswig-Holstein, Kiel, Germany

**Keywords:** heart failure, implantable cardioverter-defibrillator, depression, quality of life, PHQ-9

## Abstract

**Background:**

Heart failure (HF) is associated with development of depressive symptoms and reduced quality of life (QoL). Patients with HF and an implantable cardioverter-defibrillator (ICD) were evaluated regarding depressive symptoms and QoL.

**Methods:**

The present study included 446 patients with HF and an ICD. Depressive symptoms were assessed using the Patient Health Questionnaire 9 (PHQ-9), QoL was evaluated using the Minnesota Living with Heart Failure Questionnaire (MLHFQ). Functional ability and exercise tolerance were assessed at inclusion and after 6 months with help of the 6-min walking test (6MWT).

**Results:**

Patients included in the study had a mean age of 65.8 years and were predominantly male (83.6%), with mostly ischemic (*n* = 277; 62.1%) or dilated (*n* = 150; 33.6%) cardiomyopathy. One hundred ninety-three (43.2%) patients had depressive symptoms, of whom 75 patients (16.8%) were classified as moderate to severe depression according to the PHQ-9 at baseline. Depressive symptoms were associated with low QoL independent of NYHA functional class. High NYHA functional class, high PHQ-9 score, age and body mass index (BMI) were associated with a lower 6MWT at enrollment, while depressive symptoms (expressed as higher PHQ-9 score) and age were associated with a lower 6MWT after 6 months. Patients with history of smoking and a higher BMI showed higher PHQ-9 scores after 6 months. Patients under antidepressant medication showed improved PHQ-9 score after 6 months, indicating controlled/treated depression. However, patients with low QoL at inclusion remained with low QoL after 6 months.

**Conclusion:**

Depressive symptoms correlate with low QoL and lower long-term functional status in patients with HF and an ICD. Depressive symptoms are associated with smoking and obesity, which themselves are risk factors for a poor prognosis in HF. Only a small fraction of patients with HF and ICD showing depressive symptoms receives appropriate treatment. Assessing depressive symptoms and lifestyle factors should be part of a multimodal treatment plan in patients with HF and an ICD.

## Introduction

Heart failure (HF) is a leading cause of morbidity and mortality worldwide (1–3). HF is associated with high risk for sudden cardiac death and recurrent hospitalizations (4). In recent years, development of new therapeutic agents has led to significantly better outcomes in patients with HF (5). Nevertheless, progression of the underlying disease has significant impact on the physical and psychosocial status of patients with HF (6). Implantation of an implantable cardioverter-defibrillator (ICD) for primary prevention of sudden cardiac death (SCD) is indicated in patients with reduced left ventricular function (LVEF ≤ 35%) or for secondary prevention after hemodynamically not tolerable ventricular tachycardia or aborted SCD (5, 7–9). Patients with HF (10) and/or an ICD (11, 12) frequently report depressive symptoms. Patients with HF exhibit depressive symptoms in as high as 41.9% of cases, with 28.1% showing moderate to severe depression (10). Depressive symptoms can be detected by self-rating questionnaires such as the Patient Health Questionnaire 9 (PHQ-9), and high PHQ-9 scores have been associated with low quality of life (QoL) and high risk of hospitalization (13). HF is also associated with reduced QoL and supportive care interventions have been shown to ameliorate the QoL of patients with HF (14).

In the present study, patients with HF and an ICD were prospectively evaluated regarding depressive symptoms and QoL with the intention to elucidate the prevalence of depressive symptoms in this patient cohort and to assess how QoL evolves in the course of the underlying disease. Aim of the analysis is to identify patients in need for antidepressive treatment such as psychotherapy and/ or antidepressant medication.

## Methods

Four hundred forty-six patients presenting with HF and an ICD at Hannover Heart Rhythm Center of the Department of Cardiology and Angiology at Hannover Medical School were included between 2012 and 2014. Patients had a mean age of 65.8 ± 12.1 years at inclusion, were predominantly (83.6%) male, and were evaluated at inclusion and after 6 months in a prospective non-randomized manner. The study protocol complied with the Declaration of Helsinki and was approved by the local ethics committee. All patients gave written informed consent prior to study inclusion. Baseline parameters and medical history were retrieved through hospital records. Functional ability and exercise tolerance were evaluated at inclusion and after 6 months with the 6-min walking test (6MWT) (15). Symptoms of depression were assessed using the PHQ-9 screening tool (16). PHQ-9 scores of 0–4 were classified as none to minimal depressive symptoms, PHQ-9 scores of 5–9 as mild depressive symptoms and PHQ-9 scores of 10–27 as moderate to severe depressive symptoms (16). QoL was evaluated using the Minnesota Living with Heart Failure Questionnaire (MLHFQ) (17).

A standard 12-lead ECG was performed in all patients. ECG was performed in accordance with international standards (18). Baseline parameters were recorded including underlying disease, left ventricular ejection fraction (LVEF) and body mass index (BMI) among others. Medication at time of inclusion was documented by the treating physician after looking through the patient's charts and current list of medication.

### Statistical Analysis

Statistical analysis was conducted using SPSS version 26 (IBM, Armonk, NY, USA). Categorical variables are presented as numbers and percentages and were compared among subgroups using Chi square test. For comparison of continuous variables, the two-sided *T*-test was used. ANOVA was used to compare patient groups with none-minimal, mild, and moderate-to severe depression. Regression analysis (BACKWARD method) was used to analyze potential predictors of low functional cardiac status. We used the 6MWT at inclusion as dependent variable, and PHQ-9 sum score, LVEF, BMI, NYHA functional class, age, and gender as potential predictors. Similarly, we analyzed potential predictors of 6MWT at 6 months follow up. Data are presented as mean ± standard deviation. Values of *p* < 0.05 were considered statistically significant.

## Results

Baseline characteristics of the patients included in the study are shown in Table 1. Table 2 shows the specific device-related characteristics as well as medication at time of enrollment. Of note, a high percentage of patients (39.9%) had a cardiac resynchronization therapy (CRT) device.

**Table 1:** Baseline patient characteristics (*n* = 446).

**Parameter**	***n*** **= 446**
Age (y)	65.8 ± 12.1
Male (*n*, %)	373 (83.6%)
**Underlying disease (** ***n*** **, %)**
Ischemic cardiomyopathy	277 (62.1%)
Dilated cardiomyopathy	150 (33.6%)
Hypertrophic cardiomyopathy	8 (1.8%)
Other	11 (2.5%)
LVEF (%)	30.5 ± 6.9
**NYHA functional class (** ***n*** **, %)**
I	25 (5.6%)
II	255 (57.2%)
III	164 (36.8%)
IV	2 (0.4%)
Body mass index (kg/m^2^)	28.9 ± 5.1
Arterial hypertension (*n*, %)	311 (69.7%)
Type 2 diabetes mellitus (*n*, %)	118 (26.5%)
Hyperlipoproteinemia (*n*, %)	257 (57.6%)
Peripheral vascular disease (*n*, %)	51 (11.4%)
Chronic kidney disease (*n*, %)	106 (23.8%)
History of smoking (pack years)	34.2 ± 24.2
COPD (*n*, %)	53 (11.9%)
Creatinine (μmol/L)	114.6 ± 70.0
CRP (mg/L)	4.7 ± 8.5
NTproBNP (ng/mL)	2005.7 ± 6104.1
6-minute walking test (m)	438.7 ± 110.2
PHQ-9 (points) at inclusion	5.1 ± 4.9
MLWHF (points) at inclusion	27.0 ± 21.1
**ECG parameters**
**Atrial rhythm (** ***n*** **, %)**
Sinus rhythm	318 (71.3%)
Atrial fibrillation	106 (23.8%)
Other	22 (4.9%)
Heart rate (bpm)	67.9 ± 12.9
QTc interval (ms)	450.4 ± 44.4

*LVEF, left ventricular ejection fraction; NYHA, New York Heart Association; COPD, chronic obstructive pulmonary disease; CRP, C-reactive protein; PHQ-9, Patient Health Questionnaire; MLWHF, Minnesota Living With Heart Failure questionnaire*.

**Table 2:** Basic ICD characteristics and medication.

**ICD characteristics**
**Indication (** ***n*** **, %)**
Primary prevention	335 (75.1%)
Secondary prevention	108 (24.2%)
Unknown	3 (0.7%)
**Type of ICD (** ***n*** **, %)**
Single chamber	145 (32.5%)
Dual chamber	122 (27.4%)
Cardiac resynchronization therapy	178 (39.9%)
Subcutaneous ICD	1 (0.2%)
**Medication**
ACEI or ARB	415 (93.0%)
Beta-blockers	409 (91.7%)
Mineralocorticoid receptor antagonists	258 (57.8%)
Digitalis	77 (17.3%)
Calcium antagonists	59 (13.2%)
Sotalol	10 (2.2%)
Amiodarone	53 (11.9%)
Statin	307 (68.8%)
Antidepressant agents	27 (6.1%)

*ACE, Angiotensine-converting enzyme inhibitor; ARB, Angiotensin II receptor blocker*.

### Depressive Symptoms and Quality of Life

One hundred ninety-three patients (43.2%) showed at least mild depressive symptoms: 75 patients (16.8%) had moderate to severe depressive symptoms at baseline and 118 patients (26.5%) had mild depressive symptoms at baseline. Three groups were formed, patients with no or minimal depressive symptoms (*n* = 253), patients with mild symptoms (*n* = 118), and patients with moderate to severe symptoms (*n* = 75). Table 3 shows the assessed clinical factors examined between the three groups according to the severity of depressive symptoms.

**Table 3:** Baseline characteristics according to the severity of depressive symptoms.

	**Depressive symptoms**			
	**None-minimal (*n* = 253)**	**Mild (*n* = 118)**	**Moderate-severe (*n* = 75)**	***p*** **-value**
Age (y)	66.2 ± 11.2	66.0 ±13.1	63.8 ± 13.1	n.s.
Body mass index(kg/m^2^)	28.8 ± 4.7	28.9 ± 5.4	29.4 ± 5.9	n.s.
Heart rate (bpm)	65.5 ± 10.6	67.2 ± 10.0	74.8 ± 62.6	n.s.
6-Min-Walking Test (m)	455.7 ± 100.1	420.1 ± 112.9	407.8 ± 128.6^1^	0.042
Corrected QT intervall (ms)	448.8 ± 44.0	454.2 ± 46.9	450.1 ± 41.6	n.s.
Kreatinin (μmol/L)	110.0 ± 70.8	125.0 ± 68.7	113.9 ± 68.7	n.s.
CRP (mg/L)	4.3 ± 6.1	4.7 ± 6.6	6.3 ± 15.4	n.s.
NTproBNP (ng/mL)	2063.5 ± 7575.1	2073.5 ± 3733.6	1703.7 ± 2548.9	n.s.
PHQ-9 (points) at inclusion	1.8 ± 1.4	6.7 ± 1.4	13.9 ± 3.4^1, 2^	<0.001
MLWHF total score at inclusion	15.3 ± 13.2	34.9 ±17.8	53.5 ± 17.3^1, 2^	<0.001
Smoking (pack years)	36.7 ± 24.7	29.2 ± 17.2	33.4 ± 30.1	n.s.

*The 6-Min-Walking test (6MWT) and quality of life, assessed with the Minnesota living with heart failure scale (MLWHF) were significantly different between the groups. Particularly patients with moderate to severe depressive symptoms had a lower 6MWT compared to patients with none to minimal depressive symptoms, and reduced quality of life compared to both other groups*.

*A p-value < 0.05 was considered significant*.

*^a^ means a significant difference between patients with moderate to severe depressive symptoms compared to patients with none to minimal depressive symptoms*.

*^b^ means a significant difference between patients with moderate to severe depressive symptoms compared to patients with none to mild to moderate depressive symptoms*.

*CRP, C-reactive protein; PHQ-9, Physical Health Questionnaire; MLWHF, Minnesota Living With Heart Failure questionnaire*.

**Figure 1:**
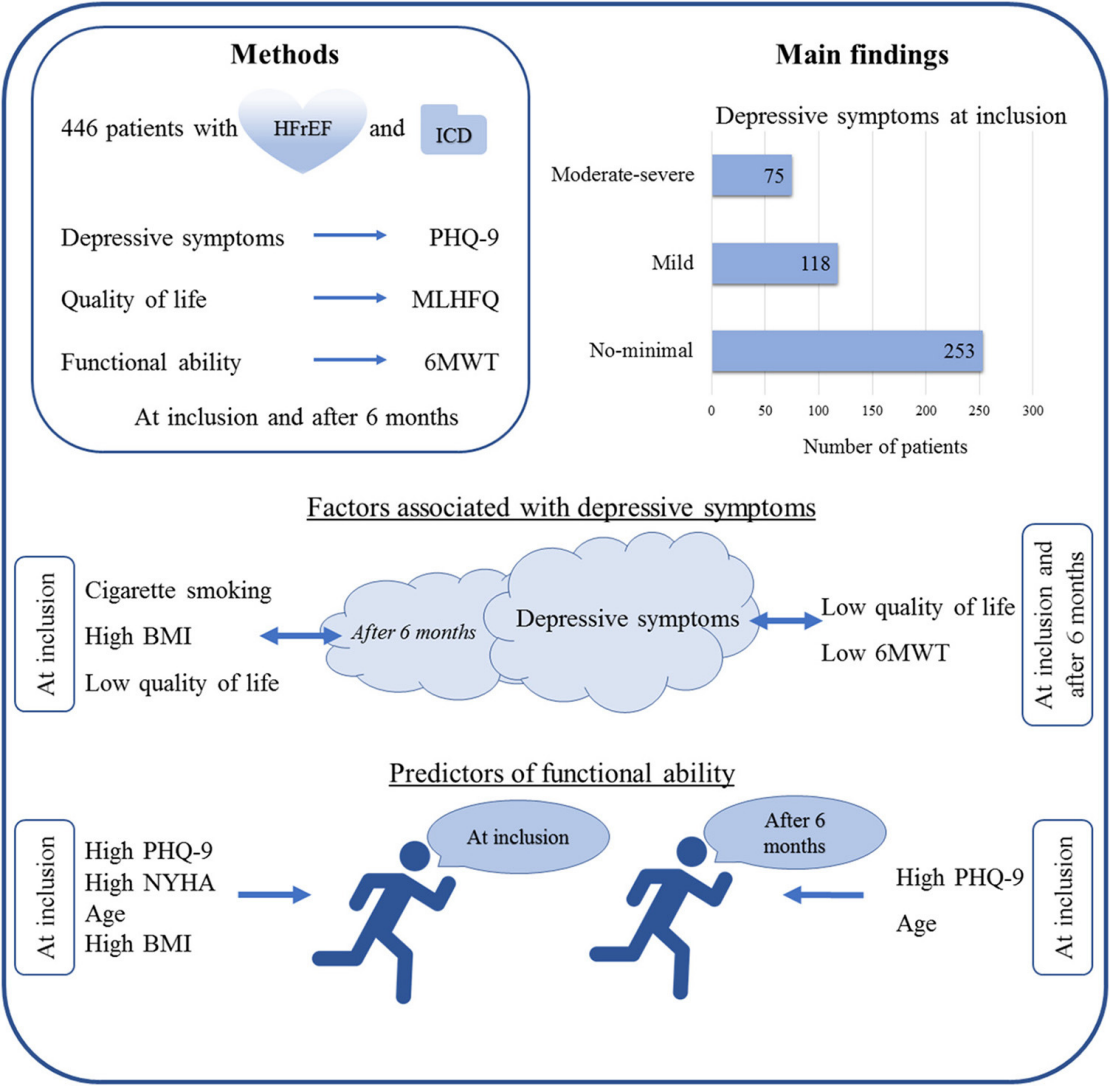
Summary figure. Overview of the methodology and main results of the present study.

Depressive symptoms at inclusion were associated with low QoL at inclusion (*p* < 0.001) and after 6 months (*p* < 0.001) independent of NYHA functional class (Figure 1).

Regression analysis (BACKWARD method) with 6MWT at inclusion as dependent variable and PHQ-9, LVEF, BMI, NYHA functional class, age, and gender as potential predictors revealed that 6MWT was predicted by PHQ-9 sum score (*p* = 0.01), NYHA functional class (*p* = 0.008), age (*p* < 0.001), and BMI (*p* < 0.001). Regression analysis (BACKWARD method) with 6MWT after 6 months as dependent variable, and PHQ-9, LVEF, BMI, NYHA functional class, age, and gender as potential predictors revealed that 6MWT was predicted by PHQ-9 sum score (*p* = 0.02) and age (*p* < 0.001) (Table 4).

**Table 4:** Overview of the results of regression analysis (BACKWARD method) regarding 6-min walking test.

	**Regression coefficient ß**	**SD**	**Beta**	***T***	***p***
**Predictors of 6-min walking test at enrollment**
Body mass index	−4.5	1.2	−0.210	−3.8	<0.001
Age (years)	−2.4	0.5	−0.274	−4.9	<0.001
NYHA functional class	−26.2	10.1	−0.142	−2.6	0.01
PHQ-9	−4.1	1.3	−0.180	−3.3	0.001
**Predictors of 6-min walking test after 6 months**
Body mass index	−2.5	1.3	−0.112	−1.8	0.076
Age (years)	−2.7	0.5	−0.298	−4.7	<0.001
PHQ-9 (points)	−3.1	1.3	−0.146	−2.4	0.02

*PHQ-9, Physical Health Questionnaire; SD, standard deviation*.

### Depressive Symptoms After 6 Months

**Figure 2:**
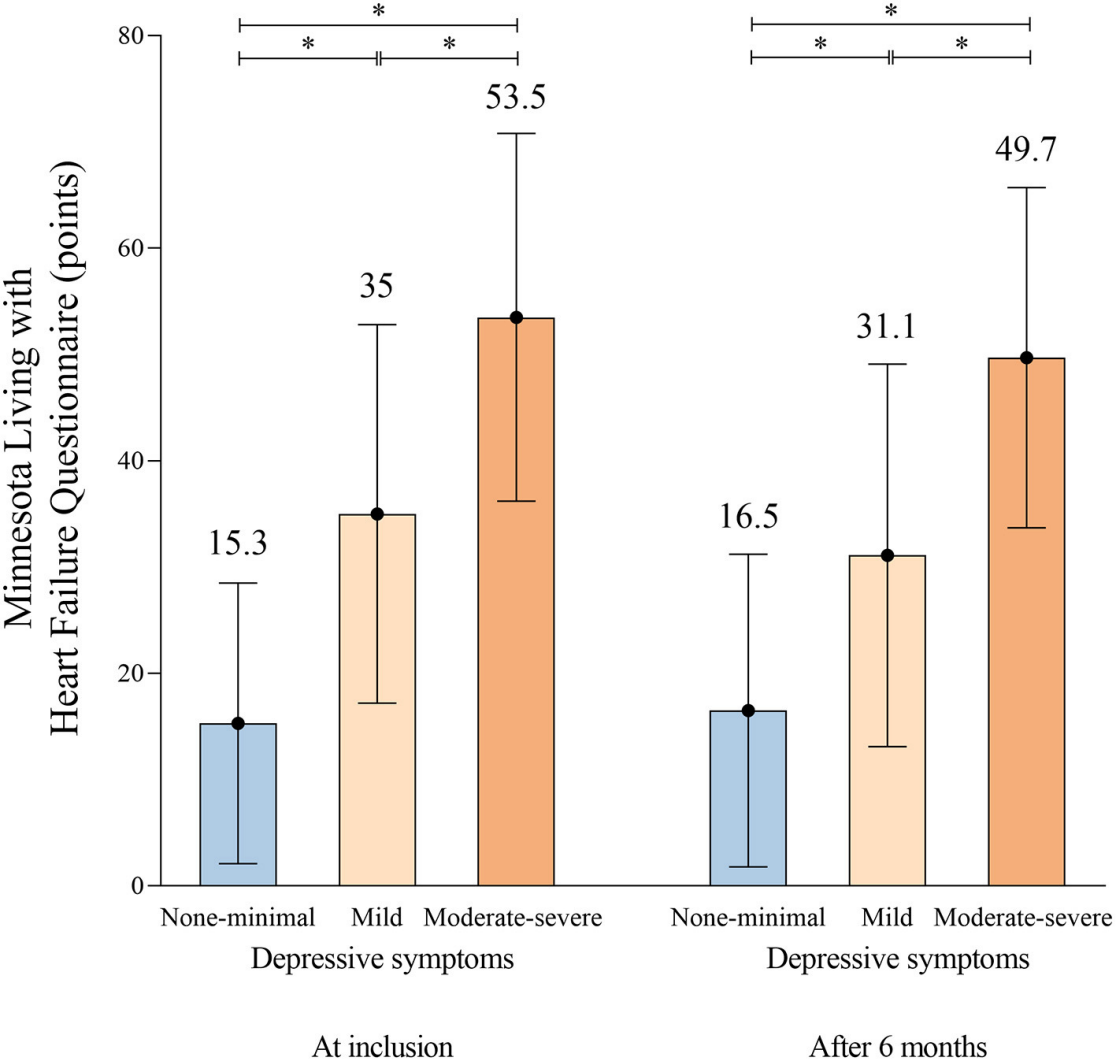
Quality of life (mean value of the Minnesota Living with Heart Failure points) according to the grade of depressive symptoms at time of inclusion and after 6 months. *p < 0.001.

Patients with high PHQ-9 score at inclusion displayed high PHQ-9 score also after 6 months (Figure 2). Patients under antidepressant medication (*n* = 27, 6.1%) showed lower PHQ-9 score after 6 months, with 12 patients showing no or minimal symptoms, 9 patients exhibiting mild symptoms and 6 patients had moderate to severe depressive symptoms. However, patients with low QoL at inclusion remained with low QoL also after 6 months. Further analysis revealed that patients with history of smoking (*p* = 0.048), a higher BMI (*p* = 0.042), low QoL at inclusion (*p* < 0.001), and low QoL after 6 months (*p* < 0.001) showed higher PHQ-9 scores after 6 months (data not shown). Furthermore, patients with a higher PHQ-9 score had a higher heart rate (*p* = 0.036).

## Discussion

The present study demonstrates the prevalence of depressive symptoms and the association with QoL in a large cohort of patients with HF and an implanted ICD. The main findings of the present study include:

1. 43.2% of patients with HF and an ICD showed mild to severe depressive symptoms. In 75 patients (16.8%), depressive symptoms were moderate to severe.
2. Depressive symptoms in patients with HF and an ICD were associated with low QoL and reduced 6MWT.
3. PHQ-9, NYHA functional class, age and BMI were predictors of low current functional ability, and PHQ-9, age, and BMI by trend were predictors of low follow-up functional ability after 6 months.
4. History of smoking, high BMI and low QoL were associated with depressive symptoms after 6 months.

These results are in accordance with data from the literature, showing high depression burden in patients with HF and ICD, and in adults with congenital heart disease (19). In a recent meta-analysis including 305,407 patients with HF, Moradi et al. reported a prevalence of 28.1% of moderate to severe depressive symptoms in this patient cohort (10). The different results between our study and the meta-analysis by Moradi et al. may be explained by the heterogeneity of the sample in the meta-analysis. Moradi et al. included HF samples from all over the world, including the EMRO region, and from countries with a lower socioeconomic level. One result of the study is that depression rates are higher in countries of the EMRO region, and in countries with lower socioeconomic level. In contrast, our study group consisted of patients from a German referral center and depression rates in our study are similar to those observed by Moradi et al. in Sweden and Italy. Bhatt et al. also reported depression rates of 26% up to 50.7% in 308 patients with HF. In the present study, 43.2% of the patients hat at least mild depressive symptoms while 16.8% of HF patients presented with moderate to severe depressive symptoms. Twenty-seven patients (6.1%) were under antidepressant medication, which could partially explain the slightly reduced rates of depressive symptoms compared to previous published studies. Furthermore, patients with multiple comorbidities, such as obesity and smoking, were more likely to exhibit depressive symptoms, a fact that has not been reported in most of the studies including patients with HF (20). Prevalence of depression also differs according to the socioeconomic level of the country in which a study is conducted (21). In general, the significant discrepancies in the reported rates of depression in patients with HF ranging from 9 to 43% (11) could be also attributed to the different instruments utilized to evaluate presence of depressive symptoms (PHQ-9, HADS etc.).

Previous studies have shown that patients with ICD show high rates (23.6%) of depressive symptoms (11), especially in case of ICD shocks. Delivery of shock therapy has an adverse impact on depression and vice versa (22, 23). Thus, therapy of depressive symptoms should be an essential part of the therapeutic strategy in patients with HF and/or ICD. In the present study, patients under antidepressant medication showed low PHQ-9 score possibly indicating controlled or treated depression. A recent work from Peng et al. could demonstrate that cognitive behavioral therapy significantly ameliorates depressive symptoms in 480 patients with HF (24).

In the present study, a high percentage of patients (*n* = 178, 39.9%) were implanted with a CRT device. CRT can improve psycho-cognitive performance in patients with HF (12). Depressive symptoms at follow-up were associated with smoking and high BMI at time of enrollment. These findings are in line with previously published data. Smoking is a well-known risk factor for depression, although patients with a genetic predisposition to depression also show higher smoking rates (25). Thus, there seems to be a bidirectional correlation between smoking and depression. Similarly, patients with a higher BMI are at high risk to develop depressive symptoms (26).

HF is associated with reduced QoL (14, 27). Also, patients with a high PHQ-9 score often show a low QoL, highlighting the impact of depressive symptoms in QoL (13). In our study, depressive symptoms were the main driver of worse QoL in patients with HF and ICD independent of NYHA functional class.

Physical and emotional support significantly improve outcomes in patients with HF (14). Nevertheless, cognitive behavioral therapy as therapeutic approach against depression did not show substantial impact on the QoL and 6MWT distance for HF (24). In the present study, depressive symptoms in ICD patients with HF were associated with low QoL. Moreover, patients with low QoL at time of inclusion remained with low QoL after 6 months. These data implicate a lack of intervention in the studied patient cohort regarding depressive symptoms and consequently QoL, although it remains unclear to which extent a therapeutic approach could impact outcomes in patients with HF. Regarding ICD and QoL, numerous studies have demonstrated that shock therapy is associated with higher morbidity and mortality (28).

### Limitations

The present study has several limitations. Since the study included patients from a single academic hospital of an urban area, it is unclear to which extent the reported depression rates allow generalizability. To which extent the duration of heart failure plays a role in depressive symptoms is unclear, since time point of first diagnosis of heart failure data were not available for most of the patients. Since patients were included between 2012 and 2014, the impact of more recent therapeutic agents such as Angiotensin-Receptor-Neprilysin inhibitors (ARNI) and Sodium/glucose cotransporter type 2 inhibitors could not be evaluated. Moreover, assessment of depressive symptoms and QoL was performed at time of enrollment and after 6 months without longer follow-ups. In this period, we observed very few major clinical events, making a correlation between depressive symptoms or QoL and major endpoints impossible. Future studies with longer follow-ups and thus more clinical events could elucidate a possible association. Depressive symptoms were evaluated with help of the PHQ-9, which represents a screening tool and should not misinterpreted as diagnostic method for depression.

## Conclusion

Patients with HF and an ICD presenting depressive symptoms have a lower QoL as well as a lower functional status according to 6MWT. Smoking, obesity and low QoL are predictors of depressive symptoms in this patient cohort. Only few patients with HF and ICD exhibiting depressive symptoms receive appropriate treatment. Thus, evaluation of depressive symptoms and lifestyle factors should be part of a multimodal treatment plan in patients with HF and an ICD.

## Data Availability Statement

The raw data supporting the conclusions of this article will be made available by the authors, without undue reservation upon reasonable request.

## Ethics Statement

The studies involving human participants were reviewed and approved by Ethics Committee of Hannover Medical School. The patients/participants provided their written informed consent to participate in this study.

## Author Contributions

CZ and KK contributed equally in conducting the analysis and writing the manuscript. DD designed the study, collected the data, conducted the analyses, supervised the statistical analyses, and wrote the manuscript. All authors made substantial contributions to the conception or design of the work, the acquisition, interpretation of data for the work, drafting the work or revising it, and approved the final version to be accountable for all aspects of the work.

## Conflict of Interest

CZ received travel grants and a fellowship grant from Biotronik and Medtronic. SH received a fellowship grant from Boston Scientific. CV received lecture honorary, travel grants advisory board fees from Bayer, Biotronik, BMS, Boston Scientific, CVRx, Daiichi Sankyo, Medtronic, Abbott, and Zoll. DD received lecture honorary, travel grants and/or a fellowship grant from Abbott, Astra Zeneca, Bayer, Biotronik, Boehringer Ingelheim, Boston Scientific, Bristol Myers Squibb, Medtronic, Microport, Pfizer, and Zoll. The remaining authors declare that the research was conducted in the absence of any commercial or financial relationships that could be construed as a potential conflict of interest.

## Publisher's Note

All claims expressed in this article are solely those of the authors and do not necessarily represent those of their affiliated organizations, or those of the publisher, the editors and the reviewers. Any product that may be evaluated in this article, or claim that may be made by its manufacturer, is not guaranteed or endorsed by the publisher.
